# Integrated antibody and cellular immunity monitoring are required for assessment of the long term protection that will be essential for effective next generation vaccine development

**DOI:** 10.3389/fimmu.2023.1166059

**Published:** 2023-11-23

**Authors:** Eustache Paramithiotis, Christophe Varaklis, Stephane Pillet, Shahin Shafiani, Mary Pat Lancelotta, Steve Steinhubl, Scott Sugden, Matt Clutter, Damien Montamat-Sicotte, Todd Chermak, Stephanie Y. Crawford, Bruce L. Lambert, John Mattison, Robert L. Murphy

**Affiliations:** ^1^ Research and Development, CellCarta, Montreal, QC, Canada; ^2^ Regulatory and Government Affairs, CellCarta, Montreal, QC, Canada; ^3^ McGill University Health Center, Montreal, QC, Canada; ^4^ Adaptive Biotechnologies, Seattle, WA, United States; ^5^ Purdue University, West Lafayette, IN, United States; ^6^ PhysIQ, Chicago, IL, United States; ^7^ Medical and Scientific Affairs, Infectious Diseases, Cepheid, Sunnyvale, CA, United States; ^8^ Department of Pharmacy Systems, Outcomes and Policy, University of Illinois Chicago, Chicago, IL, United States; ^9^ Department of Communication Studies, Institute for Global Health, Northwestern University, Evanston, IL, United States; ^10^ Health Technology Advisory Board, Arsenal Capital, New York, NY, United States; ^11^ Robert J. Havey, MD Institute for Global Health, Northwestern University, Chicago, IL, United States

**Keywords:** COVID, COVID-19, vaccine development, antibody, cellular immunity

## Abstract

The COVID pandemic exposed the critical role T cells play in initial immunity, the establishment and maintenance of long term protection, and of durable responsiveness against novel viral variants. A growing body of evidence indicates that adding measures of cellular immunity will fill an important knowledge gap in vaccine clinical trials, likely leading to improvements in the effectiveness of the next generation vaccines against current and emerging variants. In depth cellular immune monitoring in Phase II trials, particularly for high risk populations such as the elderly or immune compromised, should result in better understanding of the dynamics and requirements for establishing effective long term protection. Such analyses can result in cellular immunity correlates that can then be deployed in Phase III studies using appropriate, scalable technologies. Measures of cellular immunity are less established than antibodies as correlates of clinical immunity, and some misconceptions persist about cellular immune monitoring usefulness, cost, complexity, feasibility, and scalability. We outline the currently available cellular immunity assays, review their readiness for use in clinical trials, their logistical requirements, and the type of information each assay generates. The objective is to provide a reliable source of information that could be leveraged to develop a rational approach for comprehensive immune monitoring during vaccine development.

## Introduction

Human immune responses include a range of cellular and soluble factors. To optimally assess immune responsiveness an integrated approach that measures both cellular and soluble factors is required. However, only the antibody response was measured at scale throughout clinical development of the first generation COVID vaccines, in particular titers of neutralizing antibodies and total immunoglobulin G (IgG). Cellular immunity was partially characterized, and only at the early-stage trials (1–9). This approach left open questions about how long-term protection can be established and monitored, particularly in the context of emerging variants (10). For most vaccines antibodies can serve as adequate surrogates of general immune responses when applied to the overall population (11). In COVID, neutralizing antibody (nAb) levels have been shown to be predictive of protection from symptomatic infection in healthy, previously unexposed individuals (12, 13) though the correlate calculations are still in progress (14–16). Other evidence indicates that protection correlates may also involve non-neutralizing antibodies, as well as T cells and innate immunity (17–19). In high-risk populations, such as the elderly and people with compromised immune function, antibody measurements alone have been insufficient metrics of responsiveness to vaccination (20, 21). In addition, given how short the interval between exposure to infection can be for respiratory viruses, perhaps the most effective measures of immune responsiveness may be correlates of infection clearance and durable long term protection (22), which are functions of the cellular immune response.

## Cellular immune monitoring assays currently used in clinical trials

**Figure 1:**
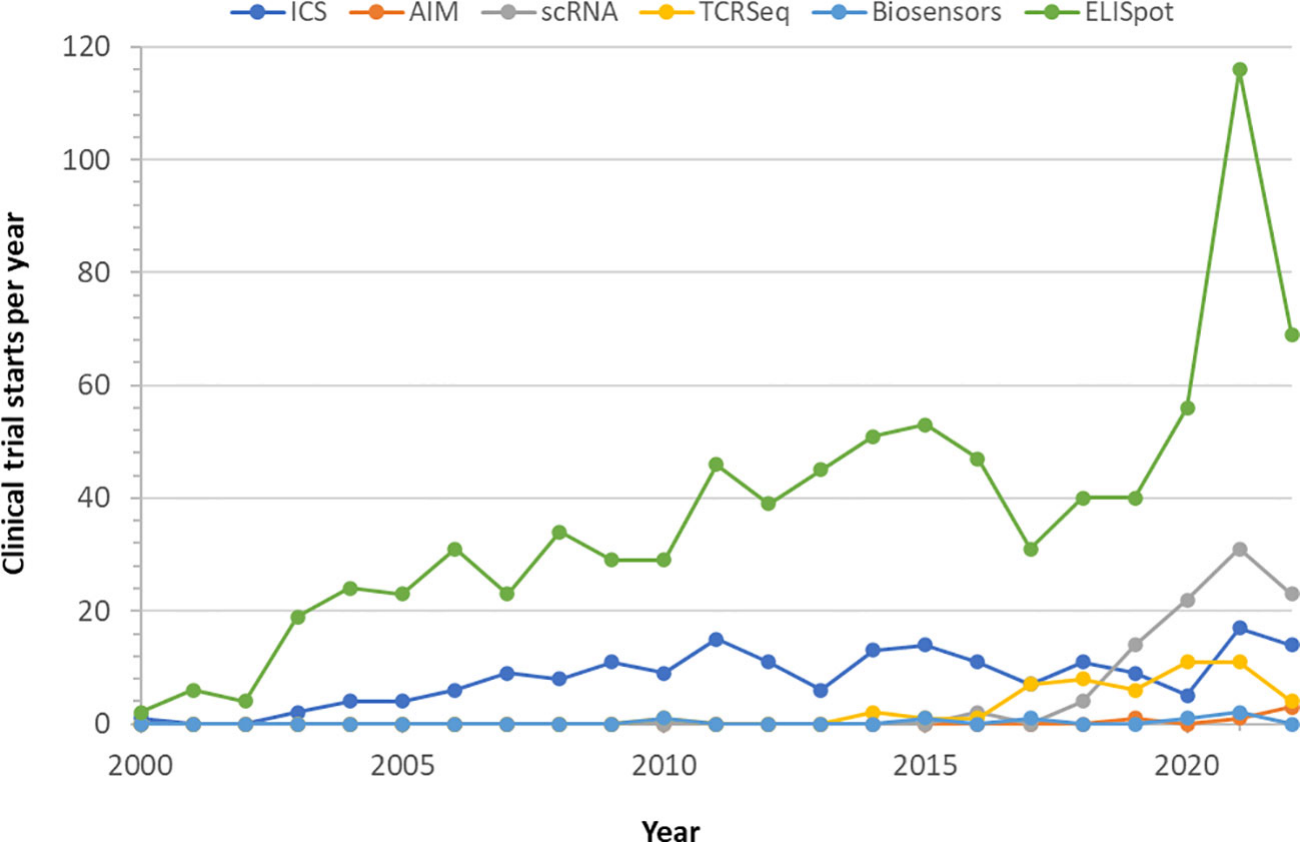
Utilization of cellular immunity monitoring assays in clinical trials. Clinical trials using one of the indicated technologies were retrieved from clinicaltrials.gov till October 2022 and displayed by start year. Completed studies and those in progress were used, withdrawn and terminated studies were excluded.

Cellular immunity is currently being measured in clinical trial settings using a number of approaches. The enzyme-linked immune absorbent spot assay (ELISpot (23); see **Box 1**) and the intracellular cytokine stain assay (ICS (24); **Box 1**) assay have both been used in clinical trials for either primary, secondary or exploratory endpoints in comparable proportions for the last twenty years (**Figure 1**). The activation induced marker assay (AIM (25); **Box 1**), another flow cytometry method, was first used for clinical trial secondary endpoints beginning in 2019. All three techniques require live peripheral blood mononuclear cells (PBMC) and *in vitro* antigen-specific stimulation prior to sample analysis, have established standardization procedures, require the same level of Clinical Laboratory Improvement Amendments (CLIA) complexity, and can be rapidly scaled with existing laboratory infrastructure. ELISpot has been the primary cellular immunology assay deployed in COVID vaccine clinical trials, reflected in its proportional increased use since 2020 (see **Figure 1**). ICS and AIM require more reagents than ELISpot, though typically ELISpot requires longer culture time. Assay sensitivity is comparable in practice. Although fewer cells can be used in ELISpot, the data becomes more variable at the lower end of the cell input scale which reduces that advantage resulting in comparable cell inputs used by all assays.

The main difference between ELISpot and the flow cytometry based ICS and AIM assays is the type of data generated. ELISpot is an antibody capture assay that provides one output, the enumeration of cells producing the particular cytokine targeted by the capture antibody, most frequently interferon-gamma (IFNγ). There is no further characterization of the responding cells. ICS is a multi-parametric flow cytometry assay which can also measure the frequency of cytokine-secreting cells, but also determines the responding cells’ lineages, activation states, and proportion of multi-functional responders, which defined as cells that make more than one cytokine following antigen-specific stimulation and which are highly desirable after vaccination (26). AIM assays typically use fewer parameters than ICS and focus on the detection of immune cell activation following antigen-specific stimulation by evaluating the expression of activation-associated surface markers, rather than the production of activation-associated cytokines (27). Since a balance of pro-inflammatory and anti-inflammatory cytokines is desirable in the regulation of host immune response (28), assessment of potential drug hypersensitivity reactions—including drug allergy and adverse drug reaction—can be gleaned from tests that detect cytokine secretion, especially tests such as ICS that enable the simultaneous detection of multiple, specific cytokines (29, 30). Due to the phenotypic characterization of the responding cells, ICS and AIM assays provide superior information than ELISpot on what kind of immune cells are involved in a response and therefore allow better mechanistic insights. This will be particularly useful for understanding how to induce robust, long lasting immune responses, especially in immune compromised individuals.

Box 1 **Immune monitoring assay definitions.**

**ELISpot: Enzyme-linked immune absorbent spot assay.** A method for measuring cytokines secreted by activated cells following antigen stimulation (23). The assay requires viable cells which are activated *in vitro* and the cytokines secreted are captured by antibodies pre-coated onto the surface of the culture wells. Readouts are colorimetric and specific for single analytes. A fluorescence version of this assay can measure up to three analytes.

**ICS: Intracellular cytokine stain assay**. A flow cytometry assay that measures the percentage of cytokine-producing cells following antigen stimulation and provides information about the cellular subpopulations producing either single or multiple cytokines of interest (24). The assay requires viable cells which are activated *in vitro* and then stained for cell surface lineage and activation markers and for intracellular cytokine production. Stimulated cells can be analyzed either prior to or following cellular expansion. Readouts are multiparametric flow cytometry files.

**AIM: Activation induced marker assay.** A flow cytometry assay that measures the percentage of activated cells following antigen stimulation and provides information about the cellular subpopulations expressing activation markers (25). The assay requires viable cells which are activated *in vitro* and then stained for cell surface lineage and activation markers Stimulated cells can be analyzed either prior to or following cellular expansion. Readouts are multiparametric flow cytometry files.

**ScRNA: Single-cell RNA sequencing assay.** A method of sequencing a cell’s complement of RNA molecules that provides high resolution information about gene expression levels in each cell analyzed as well as presence of transcriptional alterations such as alternative splicing and transcription starting site changes (31). The assay uses suspensions of cells not necessarily required to be viable, which are partitioned into single cell suspensions through limiting dilution or fluidic sorting. Cellular RNA is then extracted, reverse transcribed into cDNA and amplified using nucleotide primers containing multiple unique barcodes used for later data deconvolution and interrogation. The assay could be used on ex vivo cells or after *in vitro* stimulation. Readouts are in next generation sequencing (NGS) compatible formats.

**TCR sequencing: T cell receptor sequencing assay**. A version of scRNA analysis focused on measurement of expressed T cell receptor genes at the single cell level (32). Assay requirements and methodologies are similar to scRNA except for using amplification and data analysis methods specific for T cell receptor gene expression. Readouts are in NGS compatible formats.

**Biosensors:** Wearable biometric devices that can continuously monitor and transmit data for a range of physiologic activities such as resting heart rate, heart rate variability, dermal temperature (33), among others, and the changes observed in these measurements can be associated with immune phenomena and used as surrogate markers. These assays do not require cellular sampling from the host and provide spreadsheet compatible data in multiple formats.

A recent publication presented an overview of the technologies and differences between multiple cellular immunity assays (27), including a qualitative comparison of just the laboratory costs of running these assays. However, the establishment and maintenance of the infrastructure necessary to prepare viable PBMC of consistent quality across multiple sites is also a vital factor to consider. Once that investment is also factored in, the overall costs between cellular immunity assays become much more comparable. The differentiating feature, therefore, is the information generated by each assay, and consequently their cost effectiveness as opposed to simply cost.

## Molecular immune monitoring assays currently used in clinical trials

Single-cell RNA (scRNA) and T cell receptor (TCR) sequencing have seen a rapid rate of adoption in clinical trials in the last 5 years (**Figure 1**, **Box 1**). ScRNA allows high resolution gene expression analysis of samples at the single-cell level and is particularly suitable for studying dynamic processes such as the development, expansion or contraction of cellular immune responses, or reversible processes such as immune exhaustion (31, 34). ScRNA analysis can also be used to identify multi-functional responding cells (35, 36). TCR sequencing focuses on the antigen receptors expressed by T cells and has been used for diagnosis and personalized monitoring of remission and relapse in multiple hematologic cancers (32, 37). At the population level, TCR sequencing has been used to determine the clonality and repertoire dynamics of public T cell responses (38, 39). The information provided by these molecular methods can be expected to become relevant for assessing risk in future treatments in T cell directed vaccines such as the universal flu vaccine (40), and also likely for assessment of booster doses of existing vaccines.

## Immune monitoring assay comparison

**Figure 2:**
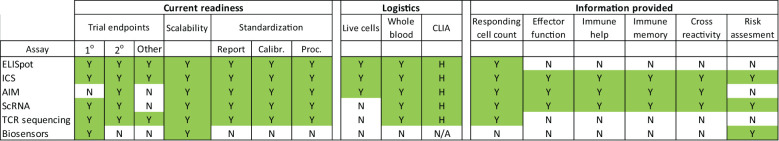
Cellular immunity assay characteristics. Color coded visualization of parameters that enable cellular immunity measurements to be conducted at clinical trial scale. Clinical trial endpoint use was determined by reviewing clinicaltrial.gov completed trials and trials in progress per assay. The highest endpoint type per trial was reported. CLIA complexity was assessed as high (H) medium (M), low (L) or non-applicable (N/A). Assay abbreviations are described in the text. Additional abbreviations not previously referred to are calibration standardization (Calibr) and procedural standardization (Proc).

A comparison of the cellular immunity assays discussed here is presented in **Figure 2**. We assessed current readiness of each assay as a composite of whether each assay had been previously used for clinical trial endpoints, plus the scalability of the assay platform, and whether standardization has been established. Scalability was evaluated by considering each platform’s installed base, and how quickly assay scale up can be implemented. Standardization was assessed by examining whether reporting, calibration and procedural standards have been established. Logistical considerations examined what kind of sample was required to carry out each test and the CLIA-regulated complexity requirements. Finally, the information generated by each assay was used to determine how detailed the immune monitoring was, and whether there was enough information to enable specific assessments such as the type of immune response induced, assessment of cross-reactivity and of risk for adverse effects such as drug hypersensitivity reactions via simultaneous detection of multiple, specific cytokines.

Multiple methods are available for high resolution immune monitoring (**Figure 2**). All the technologies reviewed here are already being used in clinical trials, with ELISpot and ICS having over two decades of use. All assays have a broad enough install base and sufficient throughput to be scalable for large trials. ELISpot, the flow cytometry, and the scRNA and TCR sequencing molecular methods have published standardization guidelines (41–48). All are sufficiently complex to warrant high level CLIA complexity designations (49) and therefore must be performed in CLIA laboratories by appropriately trained personnel.

The type of information provided by the cellular immunity assays is going to be essential for evaluation of next generation COVID vaccines, especially to define critically needed correlates associated with duration of protection (50). Phase II studies that incorporate in depth analysis of the cellular response in defined high risk populations such as the elderly or immune compromised will be able to provide new insights into the underlying immune mechanisms involved and better understanding of the dynamics of waning immunity for both cellular and humoral responses, as well as better risk assessment for novel viral variants. For example, the breadth of the immune response as measured by the total number of viral epitopes actively engaged by an individual may be a better measure of protection than measuring any one particular element of the immune response, such as nAb titer (51). Such analyses can become the basis of cellular immunity biomarkers that can then be deployed in Phase III studies using large scale-amenable genomics technologies (32, 52).

ELISpot provides the least in-depth characterization of the cellular immune response, whereas scRNA would provide the most in depth characterization. Multi-parametric flow cytometry as represented by the ICS and AIM assays falls somewhere in between, but nonetheless is still able to provide the critical information needed to evaluate the type of cellular immunity induced by the vaccination, as well as assess cross reactivity and risk of adverse effects. Judging from the overall operational costs involved, including sample procurement, processing, laboratory operations and subsequent data analysis, multi-parametric flow cytometry may be the most cost-effective application for the monitoring of vaccination-induced cellular immunity and identification of cellular immunity correlates.

ELISpot and the flow cytometry assays need live cells, which is a major source of logistical concern and also a source of data variance. Inconsistent cell preparations not only affect the yield of PBMC per tube of blood collected, but can also affect responsiveness in subsequent testing. Effective deployment of any of these cellular assays requires the establishment of properly trained sample collection and processing sites. Alternatives to PBMC preparation and cryopreservation such as whole blood ICS, whereby the blood is directly stimulated with the required antigens (53) could help alleviate the variable quality and stability issues. This protocol allows the freezing of cells following the stimulation step which would offer the potential of performing this technique directly at the clinical site but still requires careful training of all personnel involved. Additionally, automated platforms for PBMC processing also exist, and equipping clinical sites with these capabilities could be an alternative for isolating PBMC on the same day as the blood draw. The molecular methods do not require live blood cells and for direct ex vivo sample analyses can bypass the establishment of high quality sample processing. However, If the molecular assays are intended to be used on a subset of the cells, or following enrichment of antigen specific responses, the requirement for live cells of a consistent quality would remain. Nonetheless, processes for live cell preparation have been well-established in fields such as HIV where cellular immunity is a critical parameter of clinical trials, and multiple options are available for effective deployment.

Several studies have demonstrated that relatively limited number of subjects (20-30 individuals) are sufficient to demonstrate significant differences between vaccinated/treated groups and placebo control or baseline using ELISpot or ICS (3, 10, 54, 55). Since the number of subjects required depends on the magnitude of the expected effect sizes, elucidating differences between a vaccine/drug candidate and a commercially available ‘active’ comparator might be achieved with about 100 subjects or less (56). Clinical studies employing multi-parametric flow cytometry at this scale are already routinely performed. Vaccine trials that require a representative subset of each population being tested to undergo full immune monitoring, especially if that included the high risk elderly and immune compromised populations, would substantially improve our understanding of how to establish and maintain robust long-term protection. Expanding testing to larger numbers of subjects per trial would help elucidate low frequency adverse effects of the kind typically missed in small scale trials but are present when a vaccine is rolled out to the general population. Note, however, that immune compromised subjects are not typically recruited in vaccine trials, except for HIV where the target of the vaccine is the cause of the compromised immunity. Nor do correlate calculations typically seek to compare or combine cellular immunity and antibody titer data into holistic measurements of immune responsiveness to associate with clinical outcomes.

The incremental costs of these studies could be argued that they will discourage investment in vaccine development or restrict innovative approaches to vaccine development. A mitigating strategy might be to simply require the acquisition and retention of appropriate clinical samples during early developmental efforts while allowing deferral of the investment analyzing those samples until a later phase of clinical trials but prior to FDA approval. We would also recommend a deliberately agile process to determining what clinical samples should be retained and what subsequent assays should be performed as we learn more about which studies are most relevant in general, and more specifically which assays are more relevant to different infectious agents. A collateral benefit of systematically including T cell assays in the assessment of vaccines is that focusing more attention on these assays will likely accelerate simplification and cost-reduction for these tests and generate novel technical approaches to obtain comparable information.

Finally, it is important to note an emerging, but completely different technologic approach to tracking individual immune response - wearable biosensors, also known as wearable biometric devices (**Box 1**). Biosensors can continuously track a range of physiologic and behavioral changes, on an individual level. Early data from primarily consumer biosensors have found that the detection of subtle physiologic changes post-vaccination relative to pre-vaccination baselines allowed an objective quantification of individual inflammatory responses (57). Some of these studies have found a direct relationship between the degree of physiologic change and humoral immunity (33, 58). Preliminary findings using medical grade wearables appear to extend these findings to include cellular immunity. This technology is not yet mature enough to have established standardization comparable to the other technologies reviewed here, however we anticipate that it will achieve that maturity within a few years since it is already being used for clinical trial primary endpoints (**Figure 1**). Wearable biosensors do not have that level of standardization yet, and their regulation will depend on the intended use, ranging from low risk, general wellness applications that do not meet the definition of a medical device as defined in Section 201(h) of the FD&C Act (59) to high-risk medical devices requiring FDA premarket approval comparable to continuous blood glucose monitoring systems. A wearable system that is designed to monitor an immune response would likely meet the definition of a medical device and thus be subject to FDA regulations and oversight as a class II or class III medical device. We believe that the deployability, non-invasiveness, and complementarity of the data collected by wearables are expected to significantly contribute to improved understanding of host response and adverse effect monitoring in the future.

## Conclusions

COVID has brought increased public attention to the consequences of insufficient immune protection, and the limitations of insufficiently monitoring cellular immunity. We have an opportunity to integrate cellular immunity and antibody testing to inform more effective vaccination strategies and develop immune monitoring tools that should be adopted for every therapy that involves the immune system. A monolithic, homogenized approach to understanding vaccine effectiveness at a total population level overlooks the opportunity to better address the specific needs of large and growing cohorts of elderly and immunocompromised sub-populations. We can now better leverage the abundance of biotech innovation to extend our ability to understand vaccine effectiveness in different subcohorts, which itself is the first of many steps towards a future of more personalized vaccinology.

## Data availability statement

The original contributions presented in the study are included in the article/supplementary materials. Further inquiries can be directed to the corresponding author.

## Author contributions

All authors listed have made a substantial, direct, and intellectual contribution to the work, and approved it for publication.

## References

[1] Jackson LA Anderson EJ Rouphael NG Roberts PC Makhene M Coler RN . An mrna vaccine against SARS-COV-2 — preliminary report. New Engl J Med (2020) 383(20):1920–31. doi: 10.1056/NEJMoa2022483 PMC7377258 32663912

[2] Mulligan MJ Lyke KE Kitchin N Absalon J Gurtman A Lockhart S . Phase I/II study of COVID-19 RNA vaccine BNT162B1 in adults. Nature (2020) 586(7830):589–93. doi: 10.1038/s41586-020-2639-4 32785213

[3] Sahin U Muik A Derhovanessian E Vogler I Kranz LM Vormehr M . Covid-19 vaccine BNT162B1 elicits human antibody and th1 T cell responses. Nature (2020) 586(7830):594–9. doi: 10.1038/s41586-020-2814-7 32998157

[4] Kinoshita H Durkee-Shock J Jensen-Wachspress M Kankate VV Lang H Lazarski CA . Robust antibody and T cell responses to SARS-COV-2 in patients with antibody deficiency. J Clin Immunol (2021) 41(6):1146–53. doi: 10.1007/s10875-021-01046-y PMC8117127 33983545

[5] Voysey M Clemens SA Madhi SA Weckx LY Folegatti PM Aley PK . Safety and efficacy of the Chadox1 ncov-19 vaccine (AZD1222) against SARS-COV-2: An interim analysis of four randomised controlled trials in Brazil, South Africa, and the UK. Lancet (2021) 397(10269):99–111. doi: 10.1016/S0140-6736(20)32661-1 33306989 PMC7723445

[6] Ramasamy MN Minassian AM Ewer KJ Flaxman AL Folegatti PM Owens DR . Safety and immunogenicity of Chadox1 nCoV-19 vaccine administered in a prime-boost regimen in young and old adults (COV002): A single-blind, randomised, controlled, phase 2/3 trial. Lancet (2020) 396(10267):1979–93. doi: 10.1016/S0140-6736(20)32466-1 PMC7674972 33220855

[7] Sadoff J Gars ML Shukarev G Heerwegh D Truyers C de Groot AM . Safety and immunogenicity of the ad26.cov2.s COVID-19 vaccine candidate: Interim results of a phase 1/2a, double-blind, randomized, placebo-controlled trial. medRxiv (2020). doi: 10.1101/2020.09.23.20199604

[8] Liu Y Yan L-M Wan L Xiang T-X Le A Liu J-M . Viral dynamics in mild and severe cases of covid-19. Lancet Infect Dis (2020) 20(6):656–7. doi: 10.1016/S1473-3099(20)30232-2 PMC7158902 32199493

[9] Xia S Duan K Zhang Y Zhao D Zhang H Xie Z . Effect of an inactivated vaccine against SARS-COV-2 on safety and immunogenicity outcomes. JAMA (2020) 324(10):951. doi: 10.1001/jama.2020.15543a 32789505 PMC7426884

[10] Mateus J Dan JM Zhang Z Rydyznski Moderbacher C Lammers M Goodwin B . Low-dose mRNA-1273 COVID-19 vaccine generates durable memory enhanced by cross-reactive T cells. Science (2021) 374. doi: 10.1126/science.abj9853 PMC8542617 34519540

[11] Plotkin S . Correlates of protection induced by vaccination. ASM Journals (2010) . 17:1055. doi: 10.1128/cvi.00131-10 PMC2897268 20463105

[12] Khoury DS Cromer D Reynaldi A Schlub TE Wheatley AK Juno JA . Neutralizing antibody levels are highly predictive of immune protection from symptomatic SARS-COV-2 infection. Nat Med (2021) 27(7):1205–11. doi: 10.1038/s41591-021-01377-8 34002089

[13] Gilbert PB Montefiori DC McDermott AB Fong Y Benkeser D Deng W . Immune correlates analysis of the mRNA-1273 COVID-19 vaccine efficacy clinical trial. Science (2022) . 375(6576):43–50. doi: 10.1126/science.abm3425 34812653 PMC9017870

[14] Zier B Brown B Henwood D . Preparing dental hygiene students for community dental health- an interagency approach. J Can Dent Assoc (1987) 53(3):187–9. 3548923

[15] Earle KA Ambrosino DM Fiore-Gartland A Goldblatt D Gilbert PB Siber GR . Evidence for antibody as a protective correlate for covid-19 vaccines. Vaccine (2021) 39(32):4423–8. doi: 10.1016/j.vaccine.2021.05.063 PMC8142841 34210573

[16] Feng S Phillips DJ White T Sayal H Aley PK Bibi S . Correlates of protection against symptomatic and asymptomatic SARS-COV-2 infection. Nat Med (2021) 27(11):2032–40. doi: 10.1038/s41591-021-01540-1 PMC8604724 34588689

[17] Sadarangani M Marchant A Kollmann TR . Immunological mechanisms of vaccine-induced protection against COVID-19 in humans. Nat Rev Immunol (2021) 21(8):475–84. doi: 10.1038/s41577-021-00578-z PMC8246128 34211186

[18] Scurr MJ Lippiatt G Capitani L Bentley K Lauder SN Smart K . Magnitude of venous or capillary blood-derived SARS-cov-2-specific T cell response determines COVID-19 immunity. Nat Commun (2022) 13(1). doi: 10.1038/s41467-022-32985-8 PMC9492763 36130936

[19] Brasu N Elia I Russo V Montacchiesi G Stabile SA De Intinis C . Memory CD8+ T cell diversity and B cell responses correlate with protection against SARS-COV-2 following mrna vaccination. Nat Immunol (2022) 23(10):1445–56. doi: 10.1038/s41590-022-01313-z 36138186

[20] Paramithiotis E Sugden S Papp E Bonhomme M Chermak T Crawford SY . Cellular immunity is critical for assessing COVID-19 vaccine effectiveness in immunocompromised individuals. Front Immunol (2022) 13. doi: 10.3389/fimmu.2022.880784/full PMC9179228 35693815

[21] Kreuzberger N Hirsch C Andreas M Böhm L Bröckelmann PJ Di Cristanziano V . Immunity after COVID-19 vaccination in people with higher risk of compromised immune status: A scoping review. Cochrane Database Systematic Rev (2022). doi: 10.1002/14651858.CD015021 PMC9361430 35943061

[22] Willyard C . The Omicron wave's surprising lessons for long-term immunity. Nature (2022) 602:2–25. doi: 10.1038/d41586-022-00214-3 35110764

[23] Ranieri E Popescu I Gigante M . CTL ELISPOT assay. Methods Mol Biol (2014) 1186:75–86. doi: 10.1007/978-1-4939-1158-5_6 25149304

[24] Foster B Prussin C Liu F Whitmire JK Whitton JL . Detection of intracellular cytokines by flow cytometry. Curr Protoc Immunol (2007) 78(1):24.1–24.21. doi: 10.1002/0471142735.im0624s78 18432993

[25] Bowyer G Rampling T Powlson J Morter R Wright D Hill A . Activation-induced markers detect vaccine-specific CD4+ T cell responses not measured by assays conventionally used in clinical trials. Vaccines (2018) 6(3):50. doi: 10.3390/vaccines6030050 30065162 PMC6161310

[26] Dinarello CA . Historical insights into cytokines. Eur J Immunol (2007) 37:(S1). doi: 10.1002/eji.200737772 PMC3140102 17972343

[27] Schwarz M Mzoughi S Lozano-Ojalvo D Tan AT Bertoletti A Guccione E . T cell immunity is key to the pandemic endgame: How to measure and Monitor it. Curr Res Immunol (2022) 3:215–21. doi: 10.1016/j.crimmu.2022.08.004 PMC9434079 36065205

[28] Jiang P Zhang Y Ru B Yang Y Vu T Paul R . Systematic investigation of cytokine signaling activity at the tissue and single-cell levels. Nat Methods (2021) 18(10):1181–91. doi: 10.1038/s41592-021-01274-5 PMC8493809 34594031

[29] Hammond S Thomson P Meng X Naisbitt D . *In-vitro* approaches to predict and study T-cell mediated hypersensitivity to drugs. Front Immunol (2021) 12:630530. doi: 10.3389/fimmu.2021.630530 33927714 PMC8076677

[30] Yin Y Mitson-Salazar A Prussin C . Detection of intracellular cytokines by flow cytometry. Curr Protoc Immunol (2015) 110(1). doi: 10.1002/0471142735.im0624s110 26237012

[31] Ay-Berthomieu A-S . Complete guide to understanding single-cell RNA-seq. Active Motif (2021).

[32] Joshi K Milighetti M Chain BM . Application of T cell receptor (TCR) repertoire analysis for the Advancement of Cancer Immunotherapy. Curr Opin Immunol (2022) 74:1–8. doi: 10.1016/j.coi.2021.07.006 34454284

[33] Mason AE Kasl P Hartogensis W Natale JL Dilchert S Dasgupta S . Metrics from wearable devices as candidate predictors of antibody response following vaccination against COVID-19: Data from the second TEMPREDICT study. Vaccines (2022) 10(2):264. doi: 10.3390/vaccines10020264 35214723 PMC8877860

[34] Feins S Kong W Williams EF Milone MC Fraietta JA . An introduction to chimeric antigen receptor (CAR) T-Cell Immunotherapy for Human Cancer. Am J Hematology. (2019) 94(S1):S3–S9. doi: 10.1002/ajh.25418 30680780

[35] Cano-Gamez E Soskic B Roumeliotis TI So E Smyth DJ Baldrighi M . Single-cell transcriptomics identifies an effectorness gradient shaping the response of CD4+ T cells to cytokines. Nat Commun (2020) 11(1). doi: 10.1038/s41467-020-15543-y PMC7156481 32286271

[36] Stephenson E Reynolds G Botting RA Calero-Nieto FJ Morgan MD Tuong ZK . Single-cell multi-omics analysis of the immune response in COVID-19. Nat Med (2021) 27(5):904–16. doi: 10.1038/s41591-021-01329-2 PMC8121667 33879890

[37] Hussaini MO Srivastava J Lee LW Nishihori T Shah BD Alsina M . Assessment of clonotypic rearrangements and minimal residual disease in lymphoid Malignancies. Arch Pathol Lab Med (2021) 146(4):485–93. doi: 10.5858/arpa.2020-0457-OA 34343238

[38] Emerson RO DeWitt WS Vignali M Gravley J Hu JK Osborne EJ . Immunosequencing identifies signatures of cytomegalovirus exposure history and HLA-mediated effects on the T cell repertoire. Nat Genet (2017) 49(5):659–65. doi: 10.1038/ng.3822 28369038

[39] Dalai SC Dines JN Snyder TM Gittelman RM Eerkes T Vaney P . Clinical validation of a novel T-cell receptor sequencing assay for identification of recent or prior severe acute respiratory syndrome coronavirus 2 (SARS-COV-2) infection. Clin Infect Dis (2022) 75:2079–87. doi: 10.1093/cid/ciac353 PMC9129217 35521791

[40] Vijayanand S Gomes KB Gala RP Uddin MN D’Souza MJ . Exploring the potential of T-cells for a universal influenza vaccine. Vaccines. (2020) 8(4):598. doi: 10.3390/vaccines8040598 33050614 PMC7711579

[41] Hilt E Sun YS McCloskey TW Eck S McIntosh T Grugan KD . Best practices for optimization and validation of flow cytometry-based receptor occupancy assays. Cytometry (2021) 100(1):63–71. doi: 10.1002/cyto.b.21970 33259706

[42] Van der Strate B Longdin R Geerlings M Bachmayer N Cavallin M Litwin V . Best practices in performing flow cytometry in a regulated environment: feedback from experience within the european bioanalysis forum. Bioanalysis (2017) 9(16):1253–64. doi: 10.4155/bio-2017-0093 28766359

[43] O’Hara DM Xu Y Liang Z Reddy MP Wu DY Litwin V . Recommendations for the validation of flow cytometric testing during drug development: II assays. J Immunol Methods (2011) 363(2):120–34. doi: 10.1016/j.jim.2010.09.036 20946898

[44] Green CL Brown L Stewart JJ Xu Y Litwin V Mc Closkey TW . Recommendations for the validation of flow cytometric testing during drug development: I instrumentation. J Immunol Methods (2011) 363(2):104–19. doi: 10.1016/j.jim.2010.07.004 20655313

[45] Ashoor I Najafian N Korin Y Reed EF Mohanakumar T Ikle D . Standardization and cross validation of alloreactive IFNγ ELISPOT assays within the clinical trials in organ transplantation consortium. Am J Transplant (2013) 13(7):1871–9. doi: 10.1111/ajt.12286 PMC3839289 23710568

[46] Carlson CS Emerson RO Sherwood AM Desmarais C Chung M-W Parsons JM . Using synthetic templates to design an unbiased multiplex PCR assay. Nat Commun (2013) 4(1). doi: 10.1038/ncomms3680 24157944

[47] Ching T Duncan ME Newman-Eerkes T McWhorter MM Tracy JM Steen MS . Analytical evaluation of the CLONOSEQ assay for establishing measurable (minimal) residual disease in acute lymphoblastic leukemia, chronic lymphocytic leukemia, and multiple myeloma. BMC Cancer (2020) 20(1). doi: 10.1186/s12885-020-07077-9 PMC7325652 32605647

[48] Gittelman RM Lavezzo E Snyder TM Zahid HJ Carty CL Elyanow R . Longitudinal analysis of T cell receptor repertoires reveals shared patterns of antigen-specific response to SARS-COV-2 infection. JCI Insight (2022) 7(10). doi: 10.1172/jci.insight.151849 PMC9220833 35439174

[49] Center for Devices and Radiological Health . Clia categorizations. U.S. Food and drug administration. Silver Spring, Maryland:FDA (2020). Available at: https://www.fda.gov/medical-devices/ivd-regulatory-assistance/clia-categorizations.

[50] Marks PW Gruppuso PA Adashi EY . Urgent need for next-generation covid-19 vaccines. JAMA (2023) 329(1):19. doi: 10.1001/jama.2022.22759 36484995

[51] Grifoni A Sidney J Vita R Peters B Crotty S Weiskopf D . SARS-COV-2 human T cell epitopes: Adaptive immune response against COVID-19. Cell Host Microbe (2021) 29(7):1076–92. doi: 10.1016/j.chom.2021.05.010 PMC8139264 34237248

[52] Schwarz M Torre D Lozano-Ojalvo D Tan AT Tabaglio T Mzoughi S . Rapid, scalable assessment of SARS-COV-2 cellular immunity by whole-BLOOD PCR. Nat Biotechnol (2022) 40(11):1680–9. doi: 10.1038/s41587-022-01347-6 PMC10603792 35697804

[53] De Rosa SC Cohen KW Bonaparte M Fu B Garg S Gerard C . Whole-blood cytokine secretion assay as a high-throughput alternative for assessing the cell-mediated immunity profile after two doses of an adjuvanted SARS-COV-2 recombinant protein vaccine candidate. Clin. Transl Immunology (2022) 11(1):e1360. doi: 10.1002/cti2.1360 35035955 PMC8752373

[54] Pillet S Aubin E Trepanier S Bussiere D Dargis M Poulin JF . A plant-derived quadrivalent virus like particle influenza vaccine induces cross-reactive antibody and T cell response in healthy adults. Clin Immunol (2016) 168:72–87. doi: 10.1016/j.clim.2016.03.008 26987887

[55] Ward BJ Gobeil P Seguin A Atkins J Boulay I Charbonneau P-Y . Phase 1 randomized trial of a plant-derived virus-like particle vaccine for COVID-19. Nat Med (2021) 27:1071–78. doi: 10.1038/s41591-021-01370-1 PMC8205852 34007070

[56] Ward BJ Makarkov A Séguin A Pillet S Trépanier S Dhaliwall J . Efficacy, immunogenicity, and safety of a plant-derived, quadrivalent, virus-like particle influenza vaccine in adults (18–64 years) and older adults (≥65 years): Two multicentre, Randomised phase 3 trials. Lancet (2020) 396(10261):1491–503. doi: 10.1016/S0140-6736(20)32014-6 33065035

[57] Quer G Gadaleta M Radin JM Andersen KG Baca-Motes K Ramos E . Inter-individual variation in objective measure of reactogenicity following covid-19 vaccination via smartwatches and fitness bands. NPJ Digital Med (2022) 5(1). doi: 10.1038/s41746-022-00591-z PMC9019018 35440684

[58] Gepner Y Mofaz M Oved S Yechezkel M Constantini K Goldstein N . Utilizing wearable sensors for continuous and highly-sensitive monitoring of reactions to the BNT162B2 mrna COVID-19 vaccine. Commun Med (2022) 2(1). doi: 10.1002/cti2.1360 PMC9053261 35603274

[59] Center for Devices and Radiological Health . General wellness: Policy for low risk devices - guidance. U.S. Food and Drug Administration. Silver Spring, Maryland:FDA (2019). Available at: https://www.fda.gov/regulatory-information/search-fda-guidance-documents/general-wellness-policy-low-risk-devices.

